# The Role of Androgen Receptor Expression in the Curative Treatment of Prostate Cancer with Radiotherapy: A Pilot Study

**DOI:** 10.1155/2015/812815

**Published:** 2015-02-22

**Authors:** Filip Poelaert, Charles Van Praet, Anne-Sophie Beerens, Gert De Meerleer, Valérie Fonteyne, Piet Ost, Nicolaas Lumen

**Affiliations:** ^1^Department of Urology, Ghent University Hospital, De Pintelaan 185, 9000 Gent, Belgium; ^2^Department of Pathology, Ghent University Hospital, De Pintelaan 185, 9000 Gent, Belgium; ^3^Department of Radiotherapy Oncology, Ghent University Hospital, De Pintelaan 185, 9000 Gent, Belgium

## Abstract

The androgen receptor (AR) and its signaling pathway play an important role in the development and progression of prostate cancer (PCa). In the setting of primary treatment of PCa with radiotherapy (RT), where the AR can be expected to be of more importance, studies evaluating the AR expression are lacking. The goal of this research is to evaluate AR protein expression in hormone-naive PCa patients treated by RT and investigate its possible prognostic role. Primary biopsy samples of 18 patients treated with primary RT were analyzed including the corresponding clinical information. AR protein expression of the tumor epithelium (with highest Gleason pattern) and the surrounding stroma was quantified using the Quick score for steroid receptors. The differential expression between epithelium and stroma, respectively, between tumor and normal tissue (ΔTumor − ΔBenign >2 versus ≤2), was predictive for clinical progression-free survival in the biopsy samples (*P* = 0.014). Preliminary results of this research show already a promising role of differential AR expression in predicting clinical relapse after PCa treatment with primary EBRT. Further research is needed to validate these findings. Hopefully this can lead to a better understanding of PCa evolution and eventually lead to better therapy strategies.

## 1. Introduction

The epithelial compartment of the prostate consists of basal epithelial cells, intermediate cells, neuroendocrine cells, and luminal secretory epithelial cells. Stroma, surrounding the epithelial compartment, predominantly consists of connective tissue, smooth muscle cells, and fibroblasts. In the development of the prostate, the epithelial budding is androgen-dependent and requires intricate stromal-epithelial interactions. Androgens act on the stroma to (indirectly) induce prostate epithelial outgrowth during development and further homeostasis in adulthood [1].

Prostate cells depend on androgens to stimulate growth, function, and proliferation. In the 1940s, it was shown that castration of men with prostate cancer (PCa) halted the disease progression [2]. Since then, therapy that suppresses the androgen activity has been used as a systemic treatment of advanced disease. Nowadays, androgen deprivation therapy (ADT) is also used earlier in the disease progression or in recurrent disease after definitive treatment, to reduce symptoms and prevent complications [1, 3, 4].

The AR is one of the most important gatekeepers of prostate development and physiology. Due to the importance of the AR pathway, alteration in its signaling can potentially contribute to PCa development and/or progression [5–7].

The main mechanisms for both PCa development and resistance to ADT are therefore AR dependent:

- changes in intratumoral ligand concentration (e.g., in situ synthesis/metabolism),
- AR overexpression (e.g., AR gene amplification) with increased sensitivity to low ligand levels,
- AR mutations with, for example, broadened ligand specificity, hypersensitivity,
- ligand-independent AR activation, for example, splice variants, activating mutations in the LBD, and cross talk with other signaling pathways (IGF-1, EGF),
- increased transcriptional activity due to changes in coregulatory molecules.

However, AR independent mechanisms such as the deregulation of oncogenes (e.g., bcl-2, MDM2), tumor suppressor genes (e.g., p53, PTEN), and the formation of fusion genes, have also been described [6, 8, 9].

External beam radiotherapy (EBRT) is a standard treatment for localized and locally advanced PCa [3]. The gold standard technique for EBRT is intensity-modulated radiotherapy (IMRT). In case of intermediate- or high-risk disease, the use of concomitant and adjuvant ADT has shown to be beneficial compared to RT alone [10]. Although all the underlying mechanisms are not yet fully explained, ADT can combat micrometastases during treatment and it acts as a radiation sensitizer, making the PCa cells more susceptible to the effects of exposure to ionizing radiation [11]. That is why IMRT is combined with short-term ADT (4–6 months) in case of intermediate-risk PCa and with long-term ADT (18–36 months) for high-risk disease [12]. The optimal timing and/or duration of ADT in combination with radiotherapy is still a well discussed topic in the literature [13].

In the literature, several authors have tried to evaluate the prognostic role of AR expression in PCa after initial treatment with radical prostatectomy (RP) [14, 15]. However, in the setting of primary treatment of PCa with EBRT, where the AR can be expected to be of more importance, studies evaluating the AR expression are lacking.

The goal of this research is to evaluate AR protein expression in hormone-naive PCa patients treated by EBRT with curative intent (with or without ADT). Also, to see if AR protein expression can act as a prognostic and/or predictive biomarker, before and after correction for known prognostic factors such as PSA, Gleason score, and TNM-classification.

## 2. Material and Methods

A retrospective database was created with clinical PCa information and follow-up of patients receiving primary treatment for intermediate-, high-, and very-high-risk PCa. For this pilot study a retrospective search was done in patient records for PCa patients with primary diagnosis, lymph-node dissection, and EBRT treatment at our centre, starting from patients who had their treatment more than 4 years ago. When 18 patients were found, their clinical information was coded into this database.

Approval by the local ethics committee was obtained for this study (EC UZG 2011/718).

Biochemical progression after primary EBRT was defined as a rising PSA level by 2 ng/mL or more above the posttreatment nadir (cf. Phoenix definition) [16]. Clinical recurrence was defined as the development of a biopsy proven local relapse or any lymph node, bone, or visceral metastasis shown to progress on imaging. Death due to PCa also was considered as clinical failure when a previous time point with clinical failure was not stated.

From archived formalin-fixed, paraffin embedded tissue samples 5 *μ*m slides were taken from the fragment with the most aggressive tumor site (highest Gleason grade) and deparaffinized. A first slide was stained with haematoxylin-eosin to confirm presence of tumor and Gleason pattern. A second slide was stained according to the Masson's trichrome method, to easily differentiate collagen from smooth muscle fibers. A third slide was stained with the monoclonal mouse anti-human AR antibody (Clone AR441, Dako, Glostrup, Denmark) to analyze AR expression.

Gleason score was determined according to the 2005 International Society of Urological Pathology (ISUP) recommendations [17].

AR immunohistochemistry (IHC) samples were analyzed by minimal 2 readers, blinded to each other's results and blinded to the corresponding clinical information. AR expression of the tumor epithelium (with highest Gleason pattern) and the surrounding stroma was quantified using the Quick score for steroid receptors, which evaluates the proportion of cells staining and the staining intensity of these cells [18]. When a representative portion of prostate glands on the slide were free from tumor, benign epithelial cells and benign stromal cells were also analyzed and scored in the same manner. This resulted in AR expression scores of 4 compartments: tumor epithelium (TE), tumor stroma (TS), normal epithelium (NE), and normal stroma (NS). In case of interobserver Quick score discrepancy, the mathematical average was used.

Five prostatectomy specimens were analyzed before beginning analysis of the biopsy specimen. These 5 patients treated with primary RP progressed despite secondary EBRT + ADT and died due to PCa.

Quick scores ranged 6 to 8 in tumor epithelium, 0 to 1 in stroma surrounding tumor, and 2 to 6 in stroma surrounding benign epithelium. To correct for the expression in NE and surrounding NS, a new variable was considered in further analysis (ΔTumor − ΔBenign). This is the absolute difference in expression between epithelium and surrounding stroma, this from, respectively, tumor with the highest Gleason pattern and normal tissue.

Analysis of the formatted database was performed using IBM SPSS v22.0. Next to descriptive statistics, comparative statistics were performed by means of different hypothesis tests. For categorical variables, the *χ* ^2^-test and the Fisher's exact test were used where appropriate. For continuous variables with comparison between two unpaired nonparametric samples the Mann-Whitney *U* test was used. Correlation tests and other hypothesis tests were also performed. Relapse analysis was performed using the Kaplan-Meier method and log-rank test.

A *P* value < 0.05 was considered as statistically significant. Testing was performed two-sided. Graphical representations and figures of the analysis were created using SPSS.

## 3. Results

Eighteen diagnostic tissue samples of patients treated by primary EBRT were analyzed for AR protein expression. Of these, 7 were treated with prostate only EBRT and 11 with whole pelvis EBRT.

Sixteen patients received ADT and 2 patients did not. One patient, with intermediate-risk PCa, received 6 months of concomitant/adjuvant ADT and had no relapse documented in a follow-up period of 55 months. The remainder received long-term ADT treatment. No biopsy showed perineural invasion.

Eleven patients (61%) in this group of mostly high- and very-high-risk PCa patients had documented clinical relapse. Three patients (16.67%) died during follow-up, 2 directly due to PCa and one from colorectal cancer.  Table 1 compares the different parameters between patients with clinical relapse versus no clinical relapse during follow-up (no statistical significant differences between groups).

Quick scores differed between TE and NE and TS and NS (resp., *P* = 0.001 and 0.046). AR expression in TE was higher compared to NE. On the other hand, surrounding TS had lower AR expression compared to NS. The absolute difference between epithelium and stroma (ΔTumor − ΔBenign) was highly significantly different between tumor and benign tissue (*P* < 0.001).  Table 2 compares the AR expression between patients with clinical relapse versus patients with no clinical relapse during follow-up. In one biopsy sample NS could not be evaluated.

No significant differences could be found when comparing Quick scores between the 2 groups. Only with ΔTumor − ΔBenign seen as a continuous variable, the difference was highly significant (*P* = 0.002).

**Figure 1:**
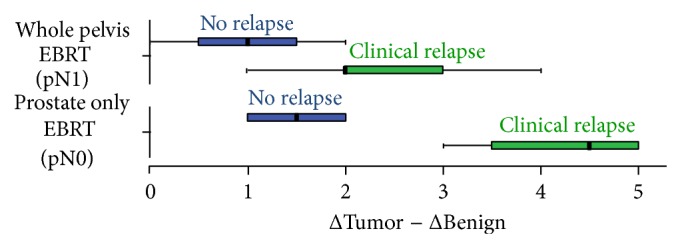
ΔTumor − ΔBenign in the relapse group versus no relapse group, stratified according to pN stage.

All patients without clinical relapse during follow-up had a ΔTumor − ΔBenign ≤2, compared to only 36.36% (*n* = 4) in the relapse group. The differential expression (ΔTumor − ΔBenign >2 versus ≤2) was associated with clinical relapse within the biopsy samples (*P* = 0.035).  Figure 1 shows the differential expression stratified according to pN stage.

Kaplan-Meier analysis was performed to identify the role of AR protein expression in predicting clinical relapse after EBRT. For stratification, the Quick score of the stroma surrounding tumor was grouped <2 (no expression) versus ≥2 (expression) and the differential expression (ΔTumor − ΔBenign) >2 versus ≤2.

In the 5 prostatectomy specimens analyzed before beginning analysis of the biopsy specimen the AR protein expression was not different between TE (the highest Gleason pattern) and NE (*P* = 0.257). However, TS had a lower expression compared to NS (*P* = 0.043). The difference in expression between epithelium and surrounding stroma was higher when comparing tumor with benign tissue (*P* = 0.039). However, no correlation could be made with prognostic factors or survival.

**Figure 2:**
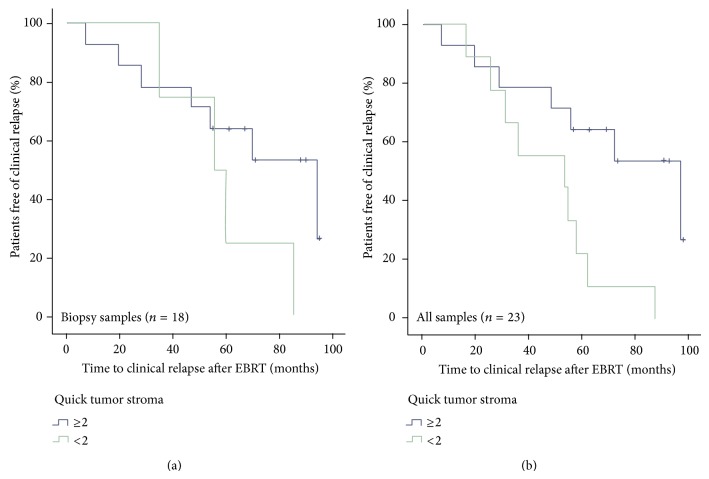
Clinical progression-free survival subdivided by Quick score of the tumor stroma <2 versus ≥2, (a) for all biopsy samples and (b) for all IHC samples (18 biopsies + 5 prostatectomy specimens).

When looking at all analyzed samples (18 biopsy + 5 prostatectomy specimens), the (loss in) expression of AR in the TS (Quick score <2 versus ≥2) was predictive for the clinical progression-free survival rates (log-rank: *P* = 0.014). Kaplan-Meier curves subdivided by TS AR expression are shown in Figure 2.

**Figure 3:**
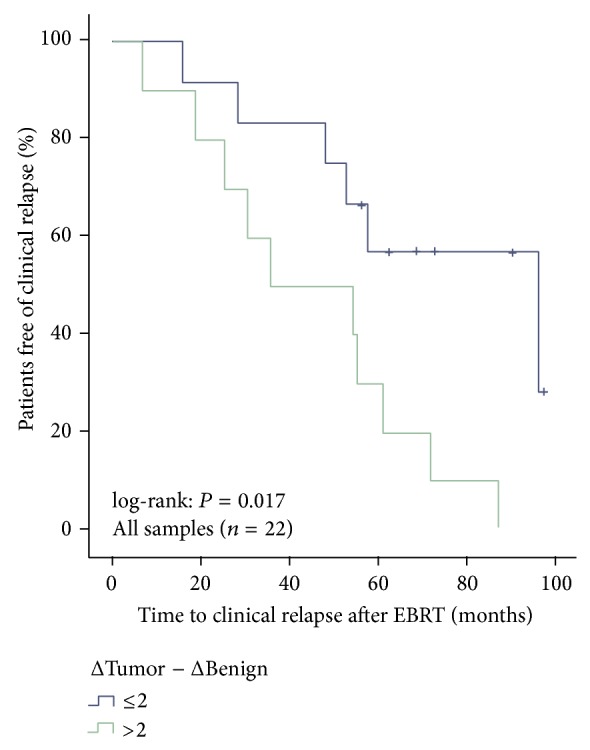
Clinical progression-free survival subdivided by ΔTumor − ΔBenign >2 versus ≤2 for all samples (1 missing value of normal stroma).

The differential expression (ΔTumor − ΔBenign >2 versus ≤2) was also predictive for the clinical progression-free survival (log-rank: *P* = 0.017) in all samples (Figure 3), however, not for overall survival after EBRT (*P* = 0.886).

**Figure 4:**
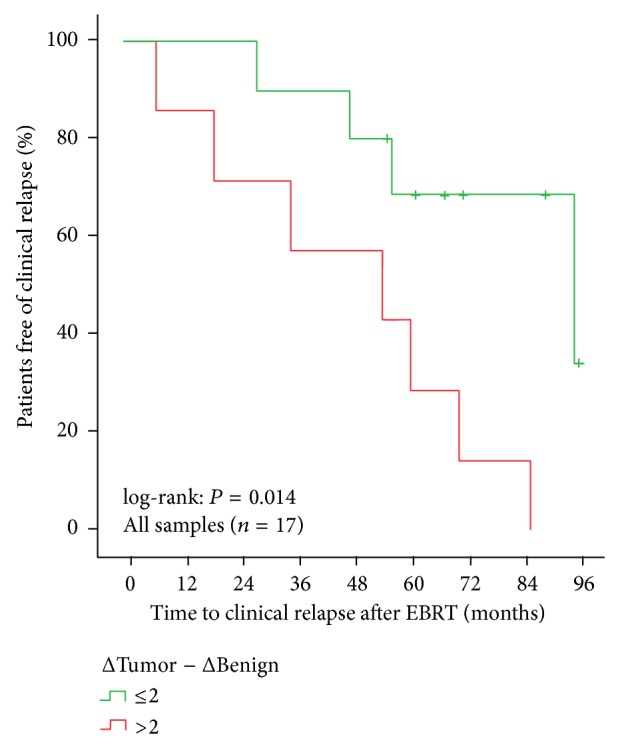
Clinical progression-free survival subdivided by ΔTumor − ΔBenign >2 versus ≤2 for all biopsy samples (1 missing value of normal stroma).

Within the biopsy samples of the primary EBRT group, only the differential expression remained significant for clinical progression-free survival (log-rank: *P* = 0.014, Figure 4).

**Figure 5:**
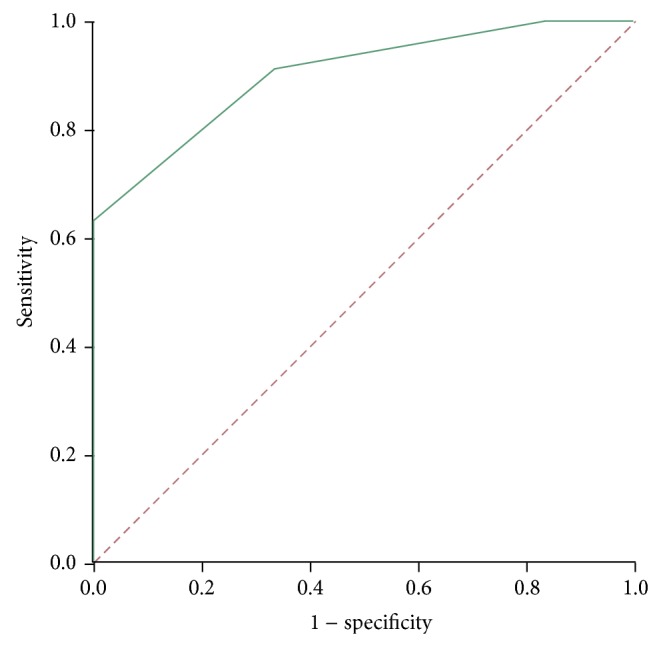
ROC-curve: representing the use of ΔTumor − ΔBenign as a prediction method for clinical relapse.

The differential AR expression between tumor and benign tissue was a good predictor for clinical relapse in the group of analyzed primary EBRT patients (AUC 0.902 [0.756–1], *P* = 0.008, Figure 5).

## 4. Discussion

Several studies have already tried to evaluate the role of AR protein in the curative treatment after RP, however, with variable results and conclusions [14, 15, 18–20]. Differences in tumor characteristics, specimens used, cohort size, techniques evaluating AR, locations of AR expression measured, and endpoints used make it very hard to compare results or to draw definitive conclusions [21].

In this research, the main focus was on primary EBRT. The benefit of adjuvant ADT to EBRT was previously proven [22]. EBRT plays an important role in PCa treatment and techniques have changed over time. A high percentage of relapse (61%) was observed due to the high percentage of high- and very-high-risk PCa patients. Due to the (very-)high-risk nature of this study population, comparison of outcome with other results in the literature has to be done carefully, taking into account all variables.

For analyzing the AR protein, several methods are available. In the setting of primary RT, PCa samples are mostly biopsy specimen. AR expression was quantified by means of IHC evaluation using the Quick score. This has proven to be a reliable and easy applicable method, mainly in breast cancer [23]. Next to evaluating the presence or absence of expression, it also enables to quantify the amount of expression (by using the proportion of cells staining combined with the staining intensity of these cells). Because of reports on the importance of the surrounding stroma (see further and also in the introduction), also stroma surrounding TE, as well as NE and stroma were scored. Using this evaluation method and using biopsy specimen, makes the results of this research relevant and reproducible in daily clinical practice.

The importance of the AR pathway in normal prostate development and functioning, as well as in PCa and further progression, is an established fact. Several studies have tried to assess the relationship between AR expression and the clinical outcome of PCa, however, with conflicting results.

Li et al. reported that high levels of AR were associated with increased proliferation and markers of aggressive disease [19]. AR expression was correlated with the cT-stage, Gleason score, and other prognostic factors such as SVI, ECE, and lymph-node invasion. This research on 640 primary RP specimen also showed that a high expression of AR was predictive for a higher probability of recurrence. After correction by multivariate analysis, it showed to be an independent prognostic indicator for biochemical recurrence-free survival [19]. Next to an increased proliferation, research shows an elevated AR protein expression in invasive PCa cells [24].

Even in CRPC cells the AR plays its role and AR expression is already elaborately researched. Knock-down of the AR decreased the serum PSA value, inhibited tumor growth, and resulted in tumor regression, in the castration resistant state [25]. AR overexpression seems to sensitize the CRPC cells to low levels of androgens [26]. The persistent AR axis output is a target for novel therapies and raised questions to introduce possible combination therapies [27].

Linja et al. reported in 2001 that AR gene amplification was present in 31% at the hormone refractory state, but not before hormone treatment. Their research showed also high expression of the AR [28]. The selective pressure exerted by ADT has been proposed to be the cause of AR gene amplification and AR mutations [29]. Merson et al., however, reported recently that focal amplification of the AR gene can be found in hormone-naive PCa. An increased gain of gene copy number was associated with a worse PCa-specific survival and suggested a possible role of early total androgen ablation [30].

These findings show that the AR is evaluated in many different settings and by many different methods. The review of Tamburrino et al. suggested that these differences could explain different results. They also suggested that the distinct evaluation of the AR status (expression and activity) in different PCa compartments may help further research [21]. In the 5 prostatectomy specimens analyzed for this report, AR protein expression was not different between TE and NE. Linking AR (over)expression to the worse prognosis of these patients is therefore difficult. In the biopsy specimens, on the other hand, AR expression was significantly higher in tumor cells compared to NE.

During prostate carcinogenesis the surrounding stroma undergoes elaborate changes by different mesenchymal-epithelial interactions and paracrine influences. The amount of smooth muscle cells reduces and cancer-associated fibroblasts appear. The hormonal PCa development seems to be independent of epithelial AR. The cross talk between tumor and surrounding stroma is still not fully understood. Stromal AR seems essential for epithelial proliferation control and is able to modulate the surrounding tumor promoting microenvironment [31–33].

Henshall et al. showed already in 2001 that the concurrent overexpression of AR in the TE and loss of AR in the surrounding stroma were associated with a poor clinical outcome in PCa after RP [20]. Ricciardelli et al. reported it to be an independent predictor of PSA relapse compared with iPSA or Gleason score [14]. This indicates the potential role of loss in AR expression of the stroma in the deregulation of prostate epithelial cell proliferation. This is contrary to the findings that a loss of stromal AR diminished the development of prostatic intraepithelial neoplasia lesions [32].

Quantification and evaluation of the expression profile of certain markers of reactive stroma have been proposed to supply additional information to other prognostic factors [34, 35]. It could assess the potential of early PCa progression [36]. Several other authors established a link between low levels of AR in tumor stroma, PCa aggressiveness, metastasis, efficacy of castration therapy, and even survival after RP [14, 18, 20, 37].

In this report, the AR protein expression also was evaluated before primary treatment with EBRT on PCa biopsy specimen. Surrounding TS had lower AR expression compared to NS (see Figure 2). Low stroma AR IHC staining has previously been related to the loss of stroma smooth muscle cells [18]. In normal tissue the AR protein expression was variable. Therefore, a new parameter was introduced (ΔTumor − ΔBenign), to correct for the AR expression profile in normal tissue. Despite absence of statistical significant differences in the distribution of several known clinical prognostic factors (see Table 1), there was interestingly a statistically significant difference for the ΔTumor − ΔBenign parameter between patients with clinical relapse versus patients without relapse during follow-up. It showed to be associated with clinical relapse-free survival.  Figure 1 suggests that this marker may be even more helpful in pN0 disease. The receiver operating characteristic (ROC) curve shows that the differential AR expression could serve as an excellent predictive marker for clinical relapse with a large area under the curve (see Figure 5).

The reciprocal interactions between neoplastic cells and supporting stromal cells are very complex. The tumor has its own microenvironment with cancer-associated fibroblasts. Epithelial-mesenchymal transition of epithelial cell enables them to invade and to resist apoptosis and to disseminate [38]. Stromal epigenetic changes can enhance the role of the AR and be sufficient to induce PCa [38].

The different therapeutic possibilities in PCa make it even more complex to understand all interactions between cancer cells and their surroundings during and after treatment. ADT has been shown to cause epithelial-mesenchymal transition in both PCa and normal prostate tissue, using a feedback loop involving the AR [39]. Arora et al. discussed the role of the glucocorticoid receptor (GR) in substituting the role of the AR after AR inhibition. Upregulation of the GR was seen after acute AR inhibition and was associated with clinical resistance to enzalutamide. They suggested that corticosteroids might even promote tumor progression when the tumor expresses the GR and this resistance mechanism [40].

Next to the AR, other potential markers have been suggested. Decreased PTEN expression has been associated with an increased risk of recurrence in patients with clinically localized PCa treated with RP, independent of known clinicopathological factors [41]. Also estrogen has been suggested to play a role in the progression of PCa. The variations in steroid-metabolizing enzymes during PCa control the bioavailability of active steroid hormones in the prostate and may be crucial in the regulation of growth [42].

Necrotic cell death releases proinflammatory signals into the surrounding tissue microenvironment, in contrast to apoptosis and autophagy [43]. Research of El-Saghire et al. showed that low doses radiation with IMRT can induce proinflammatory and prosurvival responses in PCa patients [44]. After RT, expression of the AR, but also of estrogen receptor-*α* and -*β*, seems to have increased [45]. Research of Polkinghorn et al. shows that AR signaling regulates DNA repair in PCa [11]. This can be a potential mechanism of the synergy between ADT and ionizing radiation. Adjuvant ADT to EBRT has its synergistic effects by decreased tumor cell hypoxia, decreased DNA repair, and decreased AR mediated cell growth [11].

An important shortcoming is the small sample size of analyzed AR IHC specimens. Further analysis of other available specimens and clinical data was not done for this pilot study. However, the displayed results are already very promising.

Further sample analysis is needed to validate if the differential expression of AR protein expression can be of importance in the primary EBRT setting. This research report emphasizes the role of the AR in this setting due to the link between AR, DNA repair, and ADT. The goal is to see if AR protein expression can act as a prognostic marker (of patient outcome independent of given therapy) and/or a predictive marker (of the possible response to a specific therapy) in the setting of EBRT with or without ADT [46].

The role of different hormones and the alterations in PCa progression have to be further investigated. This will help to explore benefits of different treatments and/or even play its role in personalized medicine. The interaction with stroma should be taken into account in cell culture research [47]. Targeting the tumor microenvironment and the stromal AR could be attractive as a therapeutic strategy because of its critical involvement in PCa [33, 48].

## 5. Conclusion

The setting of primary EBRT can be very interesting to evaluate the prognostic and predictive role of AR protein expression, especially due to interaction of ADT (often given concomitant/adjuvant with EBRT) and the AR. The differences in AR expression observed in this research suggest that a changed AR expression of epithelium and its surrounding stroma could be of importance.

Preliminary results show already a promising role of differential AR expression in predicting clinical relapse after treatment of PCa with primary EBRT. Further validation of these findings is necessary.

## Figures and Tables

**Table 1:** Comparison between clinical relapse group and group without relapse during follow-up.

Parameter	Clinical relapse (*n* = 11)	No relapse during follow-up (*n* = 7)

Follow-up (months, median [range])		
After diagnosis	73 [46–153]	75 [60–101]
After primary EBRT	66 [40–148]	71 [55–95]
Age at diagnosis (*y*, median [range])	64 [53–74]	63 [57–76]
Initial PSA (ng/ml, median [range])	30.1 [5.52–126.4]	13.7 [9.10–75]
PSA category (*n* (%))		
PSA <10 ng/ml	2 (18.2)	1 (14.3)
PSA 10–20 ng/ml	3 (27.3)	3 (42.9)
PSA >20 ng/ml	6 (54.5)	3 (42.9)
N+ (yes (%))	7 (63.6)	4 (57.1)
Gleason score (*n* (%))		
≤6	1 (9.1)	—
7	3 (27.3)	1 (14.3)
≥8	7 (63.6)	5 (71.4)
Gleason 5 component (yes (%))	5 (45.5)	5 (71.4)
Clinical T-stage (*n* (%))		
cT1	1 (9.1)	—
cT2	2 (18.2)	3 (42.9)
cT3	7 (63.6)	3 (42.9)
cT4	1 (9.1)	1 (14.3)
EAU risk classification (*n* (%))		
Low-risk	—	—
Intermediate-risk	1 (9.1)	1 (14.3)
High-risk	2 (18.2)	1 (14.3)
Very-high-risk	8 (72.7)	5 (71.4)

**Table 2:** Comparison of Quick scores and calculated values (median [range]).

Location	Clinical relapse (*n* = 11)	No relapse during follow-up (*n* = 7)

Tumor tissue		
Epithelium	8 [6–8]	8 [4–8]
Stroma	2 [0–7]	3 [2–5]
Benign tissue		
Epithelium	6 [0–8]	7 [6–8]
Stroma	3 [0–5]	3.5 [3–5]
Epithelium − stroma		
ΔTumor	6 [1–8]	5 [1–5]
ΔBenign	3 [−3–6]	3.5 [2–5]
ΔTumor − ΔBenign	3 [1–5]	1.5 [0–2]
ΔTumor/ΔBenign	2 [−0.33–6]	1.38 [1–2]
Tumor stroma − Benign stroma	−1 [−3–4]	−1/2 [−1–0]
Tumor stroma/Benign stroma	0.75 [0–2.33]	0.9 [0.67–1]
